# Expression of Cholera Toxin (CT) and the Toxin Co-Regulated Pilus (TCP) by Variants of ToxT in *Vibrio cholerae* Strains

**DOI:** 10.3390/toxins15080507

**Published:** 2023-08-17

**Authors:** Donghyun Lee, Hunseok Choi, Seonghyeon Son, Jonghyun Bae, Jayun Joo, Dong Wook Kim, Eun Jin Kim

**Affiliations:** 1 Department of Pharmacy, College of Pharmacy, Hanyang University, Ansan 15588, Republic of Korea; 2 Institute of Pharmacological Research, Hanyang University, Ansan 15588, Republic of Korea

**Keywords:** cholera, *Vibrio cholerae*, cholera toxin (CT), toxin co-regulated pilus (TCP), ToxT

## Abstract

The expression of the two major virulence genes of *Vibrio cholerae*—*tcpA* (the major subunit of the toxin co-regulated pilus) and *ctxAB* (cholera toxin)—is regulated by the ToxR regulon, which is triggered by environmental stimuli during infection within the human small intestine. Special culture methods are required to induce the expression of virulence genes in *V. cholerae* in the laboratory setting. In the present study, induction of the expression of virulence genes by two point mutations (65th and 139th amino acids) in *toxT*, which is produced by the ToxR regulon and activates the transcription of the virulence genes in *V. cholerae*, under laboratory culture conditions has been investigated. Each of the four *toxT* alleles assessed displayed different transcriptional activator functions in a given *V. cholerae* strain. Although the ToxR regulon has been known to not be expressed by El Tor biotype *V. cholerae* strains cultured under standard laboratory conditions, the variant *toxT* alleles that we assessed in this study enabled the expression virulence genes in El Tor biotype strains grown under simple culture conditions comprising shake culture in LB medium, suggesting that the regulation of virulence gene expression may be regulated more complexly than previously thought and may involve additional factors beyond the production of ToxT by the ToxR regulon.

## 1. Introduction

Cholera is a severe diarrheal disease caused by the Gram-negative bacterium *Vibrio cholerae* [1,2,3]. Among the more than 200 serogroups identified based on the structure of *V. cholerae* O-antigens, two serogroups—O1 and O139—have been recognized as the causative agents of epidemic cholera [1]. Bacterial strains belonging to these two serogroups produce cholera toxin (CT), which is a typical AB bacterial toxin that is composed of a single active subunit (CTA) and five binding subunits (CTB) and causes disease symptoms in the human small intestine.

The O1 serogroup *V. cholerae* strains are further divided into two biotypes—classical and El Tor—depending on their biochemical and microbiological characteristics [2]. Of the seven cholera pandemics that have been recognized since the early 19th century, the first six were presumed to have been caused by the classical biotype strains, while the El Tor biotype strains were responsible for the seventh cholera pandemic, which began in 1961 and still ongoing [4,5].

In addition to CT, the toxin co-regulated pilus (TCP) has been shown to play an important role in the intestinal colonization of cholera bacteria [6,7]. The TCP is a Type IV pilus and is mainly composed of the subunit protein TcpA [7]. During the course of human infection, the expression of two major virulence factors of *V. cholerae*, CT and the TCP, is triggered by environmental stimuli in the small intestine [8,9].

The mechanisms for the regulation of virulence gene expression in *V. cholerae* strains have been extensively studied. The expression of CT, the TCP, and other virulence factors is controlled by a cascade of regulatory proteins known as the ToxR regulon [10,11]. Environmental stimuli, such as changes in pH, oxygen concentration, temperature, etc., induce the production of TcpP/TcpH, which interacts with ToxR/ToxS. This complex promotes the production of ToxT, which is an AraC/XylS-type transcriptional regulator, that directly activates the transcription of *ctxAB* and *tcpA* [12,13,14]. 

Different laboratory conditions are known to be required to induce the expression of the virulence genes in the classical or El Tor biotype strains [15,16]. Classical biotype strains produce CT and the TCP under relatively simple laboratory culture conditions, requiring only bacterial growth at 30 °C in LB medium, with the pH adjusted to 6.5, which have also been described as agglutinating conditions [15,17]. More complicated culture methods have been developed to induce virulence gene expression in El Tor biotype strains [18,19,20]. The most frequently employed method is termed AKI conditions, under which bacteria are incubated in AKI broth (1.5% Bacto peptone, 0.4% yeast extract, 0.5% NaCl, and 0.3% NaHCO_3_) at 37 °C for 4 h under static conditions, followed by vigorous shaking for 16 h [16]. However, the virulence gene expression level varies among El Tor biotype strains, even under AKI conditions (i.e., some El Tor biotype strains produce a comparable, or greater, amount of CT than the most studied classical biotype strain O395, grown under agglutinating conditions, while others produce only a negligible amount of CT or do not produce any virulence factors at all [21,22]). 

While most *V. cholerae* strains contain an identical amino acid sequence in *toxT*, we have identified a variant of *toxT* in a Wave 2 atypical El Tor biotype strain, MG116025 [23,24]. A Tyr residue at amino acid position 139 is substituted by Phe in this variant *toxT*, which has been previously described as *toxT*-139F [25]. This allele is described as *toxT*-65S-139F, or *toxT*-SF in this report, while the *toxT* allele found in other *V. cholerae* strains that contains Tyr at the amino acid position 139 is denoted as *toxT*-65S-139Y, or *toxT*-SY (Table 1). We have introduced the *toxT*-SF allele in a number of *V. cholerae* strains, and we found that the *toxT*-SF allele can facilitate virulence gene expression in a simple shake culture in many, but not all, *V. cholerae* strains [23,25,26,27]. 

We have identified another variant *toxT* allele in a classical biotype strain, 569B, that contains Ala at amino acid position 65 (*toxT*-65A-139Y, or *toxT*-AY, Table 1) instead of the Ser that is present in other *toxT* alleles. Although strain 569B bearing the *toxT*-AY allele expresses the virulence genes, the introduction of the *toxT*-AF allele (with Phe at the 139th amino acid position) significantly enhances this virulence gene expression [27]. 

In the present study, we examined whether a specific variant of *toxT* could uniformly enhance virulence gene expression in *V. cholerae* strains (at least among the same biotype strains), given that ToxT is the direct transcriptional activator of virulence gene expression. We introduced four *toxT* alleles in multiple *V. cholerae* strains (four classical biotype strains and five El Tor biotype strains) and examined the virulence gene expression under simple laboratory culture conditions (LB medium at 30 °C or 37 °C). 

## 2. Results

### 2.1. Virulence Gene Expression Conditions

In the present study, four *V. cholerae* O1 classical biotype strains (O395, 569B, Cairo48, and Cairo50) and five El Tor biotype strains (N1696, T19479 (belonging to the Wave 1 El Tor biotype strain), B33, MG116025 (classified as a Wave 2 El Tor biotype strain), and IB5230 (a 2010 Haitian cholera outbreak strain that is grouped as a Wave 3 El Tor biotype strain)) and isogenic derivatives of each strain that contained variant *toxT* alleles, or had the *toxT* gene deleted (Δ*toxT*), were analyzed for CT/TCP expression under laboratory culture conditions (Table 2). 

We examined the culture suspension for the expression of CT by western blot image analysis using anti-CTB to analyze secreted and intracellular CT. We examined the harvested cells for the expression of the TCP by western blot image analysis using anti-TcpA. We have reported that *V. cholerae* strains could be transduced by CTXΦ when TcpA expression was elevated by the *toxT*-SF variant [23], which implies that more functional TCP were produced by the increased expression of TcpA.

When the isogenic derivatives were constructed, more than 10 independent colonies of each construct were examined for virulence gene expression, primarily in virulence-inducing conditions—in the agglutinating condition for the classical biotype strains or in the AKI condition for the El Tor biotype strains. We have confirmed that a derivative that expressed CT/TCP at the virulence-inducing culture condition always expressed CT/TCP at the same culture condition (though there were negligible variations in the CT/TCP expression level between colonies), while a non-expressing derivative was always a non-producer.

**Figure 1:**
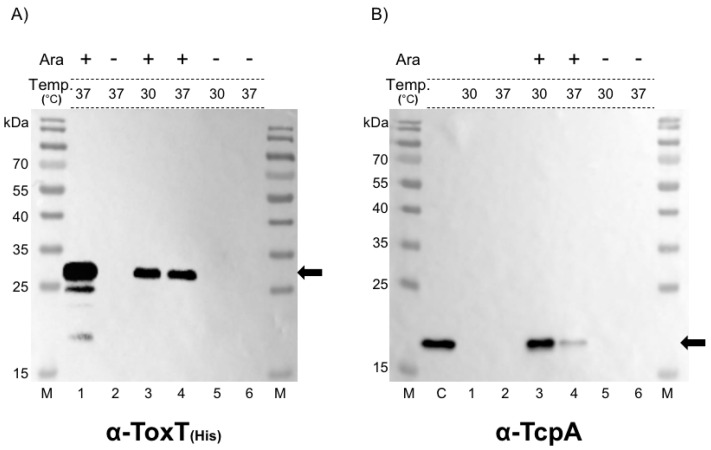
Expression of TcpA in isogenic derivatives of the classical biotype strain O395. (**A**) Expression of *toxT* under the control of the P_BAD_ promoter was analyzed by western blotting with anti-His-tag. *E. coli* DH5α harboring pBAD-toxT was cultured in the presence (lane 1) or absence (lane 2) of 0.2% arabinose in LB media at 37 °C. EJK010 (O395-Δ*toxT*) that contained pBAD-toxT was cultured at 30 °C (lane 3 and 5) or at 37 °C (lane 4 and 6) in the presence (lanes 3 and 4) or absence (lanes 5 and 6) of 0.2% arabinose. The arrow indicates ToxT-His. (**B**) Expression of TcpA by trans-complementation of pBAD-toxT in the *toxT*-deleted strain EJK010. IB5230 cultured in AKI broth at 37 °C was used as a positive control (lane C). EJK010 was cultured in LB media at 30 °C (lane 1) or at 37 °C (lane 2). EJK010 that contained pBAD-toxT was cultured in LB media at 30 °C (lanes 3 and 5) or at 37 °C (lanes 4 and 6) in the presence (lanes 3 and 4) or absence (lanes 5 and 6) of 0.2% arabinose. The arrow indicates TcpA. Coomassie brilliant blue staining of the same samples is shown in Figure S1.

**Figure 2:**
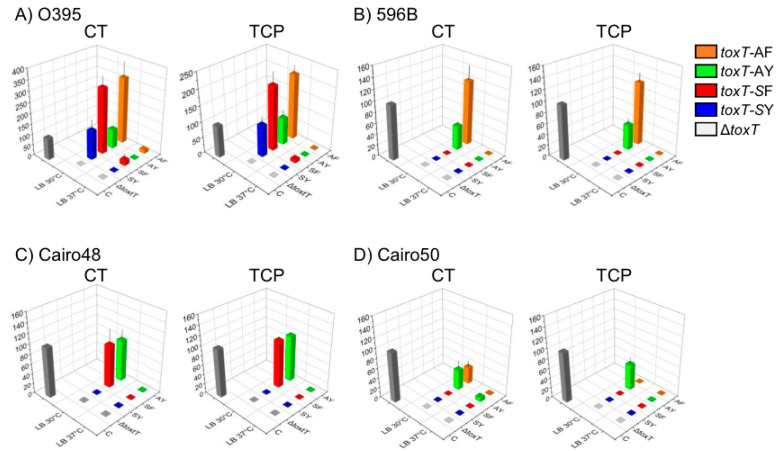
CT and TCP expression by *toxT* variants in classical biotype strains of *V. cholerae*. (**A**) O395, (**B**) 569B, (**C**) Cairo48, and (**D**) Cairo50. Relative expression of CT (right) and TCP (left) in *V. cholerae* bearing *toxT* variants (*toxT*-SY: blue, *toxT*-SF: red, *toxT*-AY: green, and *toxT*-AF: orange) cultured in LB medium at 30 °C and 37 °C was compared to the positive control (IB5230 cultured in AKI broth at 37 °C, black). Neither CT nor TCP were expressed in *toxT*-deleted strains (gray). Three independent experiments were performed. Mean ± standard deviation values are presented. Relative expression level data are presented in Table S1.

**Figure 3:**
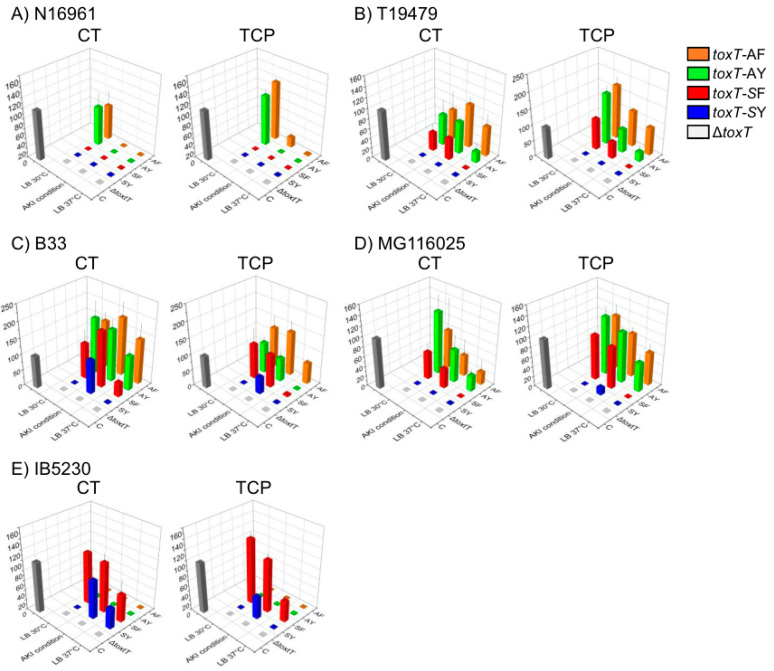
CT and TCP expression by variants of *toxT* in El Tor biotype strains of *V. cholerae*. (**A**) N16961, (**B**) T19479, (**C**) B33, (**D**) MG116025, and (**E**) IB5230. Relative expression of CT (right) and TCP (left) in *V. cholerae* bearing *toxT* variants (*toxT*-SY: blue, *toxT*-SF: red, *toxT*-AY: green, and *toxT*-AF: orange) cultured in LB medium at 30 °C and 37 °C and under AKI conditions was compared to the positive control (IB5230 cultured in AKI broth at 37 °C, black). Neither CT nor the TCP were expressed in the *toxT*-deleted strains (gray). Three independent experiments were performed. Mean ± standard deviation values are presented. Relative expression level data are presented in Table S2.

There was no CT/TCP expression in the Δ*toxT* isogenic derivative of each strain, as previously reported (Figure 1, Figure 2 and Figure 3) [32]. To confirm that the deletion of *toxT* did not affect other genes, we trans-complemented the *toxT*-deleted isogenic derivative of O395 (EJK010) by introducing a *toxT*-SY open reading frame (ORF) under the arabinose-inducible promoter in the pBAD24 plasmid (Figure 1A) [33] and verified that *tcpA* expression occurred in the trans-complemented strain only when 0.2% arabinose was added to the culture (Figure 1A,B)) [34,35]. Notably, more TcpA protein was expressed when it was cultured at 30 °C than at 37 °C in the trans-complemented EJK010, although the ToxT expression levels by the P_BAD_ promoter were similar (Figure 1B). This result suggests that, not only the production of ToxT, but also additional factor(s) that were potentially affected by temperature, play important roles in the expression of virulence genes.

In this study, we monitored CT and TcpA expression in classical biotype strains that were cultured in LB medium at 30 °C (pH not adjusted at 6.5) and in LB medium at 37 °C (Figure 2 and Figure S2), because there was no significant difference in CT or TcpA expression in the classical biotype strain O395 when cultured under agglutinating conditions or in simple LB medium at 30 °C (Figure S2) [25]. Both CT and TcpA expression were assessed in El Tor biotype strains that were cultured in LB medium at 30 °C, AKI condition, and LB medium at 37 °C (Figure 3 and Figure S3).

Previously, we examined the expression of CT and TcpA in the hypervirulent strain IB5230 cultured in AKI broth at 37 °C (without static incubation, which should be distinguished from AKI conditions) and found that the CT and TcpA expression levels were similar to those of O395 cultured in agglutinating conditions [25,27]. In this study, the CT and TcpA expression levels of the isogenic derivatives of each *V. cholerae* strain were compared to those of the IB5230 strain cultured in AKI broth at 37 °C. 

### 2.2. Classical Biotype Strains

O395: The virulence gene expression in the O395 (*toxT*-SY) strain has been extensively studied [21,34]. The *toxT*-SF allele (YJB001) increased CT expression to approximately 350% of the control values (IB5230 cultured in AKI broth at 37 °C, which was similar to O395 in the agglutinating conditions), which is consistent with the findings of our previous report [25]. However, CT expression in EJK008 (*toxT*-AY) remained similar to that in the O395 strain, while CT expression in EJK009 (*toxT*-AF) increased to more than 300% of the control value (Figure 2A). In addition, the TcpA expression pattern was similar to the CT expression in the isogenic derivatives of O395, and the TcpA expression of EJK008 was similar to that of O395, and increased by 200% in YJB001 and EJK009 [27]. Small amounts of CT (20% of the control in YJB001 and EJK009) and TcpA (10% of the control in EJK009) were expressed at 37 °C, while no other classical biotype strains or their derivatives expressed CT or TcpA at 37 °C. Notably, O395 was the only strain in which all four of the variants of *toxT* expressed CT and TcpA.

569B: The 569B strain is also one of the well-studied classical biotype strains and has been utilized in the VAXCHORA live attenuated oral cholera vaccine [36]. This strain originally contained the *toxT*-AY allele and expressed approximately 45% of the CT and TcpA expressed by the control strain (Figure 2B). The introduction of the *toxT*-AF allele (EJK007) enhanced CT and TcpA expression at 30 °C to 120% of the control (Figure 2B) [27]. Virulence gene expression at 30 °C was abolished in EJK011 and EJK0012, which contained the *toxT*-SY and the *toxT*-SF allele, respectively. No isogenic derivatives of 569B produced CT or TcpA at 37 °C (Figure 2B).

Cairo48: The Cairo48 strain has been used in inactivated oral cholera vaccines together with Cairo50 [37]. These two classical biotype strains that contain the *toxT*-SY allele did not express TcpA under agglutinating conditions, unlike the O395 strain (Figure 2C and Figure S2) [27]. The expression of both CT and TcpA was stimulated by the introduction of the *toxT*-SF (EJK003) or *toxT*-AY (EJK017) alleles when the organisms were cultured at 30 °C, similar to their expression in the O395 under agglutinating conditions (Figure 2C). Unfortunately, an isogenic derivative containing the *toxT*-AF allele could not be constructed, even after extensive construction trials. Neither CT nor TcpA were produced in the derivatives of this strain at 37 °C.

Cairo50: Cairo50 and EJK004 (*toxT*-SF) did not express CT or TcpA in agglutinating conditions, however, a small amount of TcpA was expressed in EJK004 when cultured in AKI broth at 30 °C [27]. The introduction of *toxT*-AY (EJK014) slightly stimulated CT (20% of the control) and TcpA (25% of the control) expression at 30 °C (Figure 2D), while only CT expression was marginally stimulated (20% of the control) by *toxT*-AF (EJK015). CT and TcpA were not expressed at 37 °C in the isogenic derivatives of this strain, except for the negligible amount of CT (5% of the control) in EJK014 (Figure 2D).

### 2.3. El Tor Biotype Strains

Although AKI conditions have been developed for the production of CT and TcpA in El Tor biotype strains, the capacity of virulence factor production varies between strains [22,38]. In our experimental set-ups, some of the strains that contained the *toxT*-SY allele (N16961 and T19479) did not express CT, while other strains expressed 50% (IB5230) to 200% (B33) of the CT expressed by the control strain. The introduction of the *toxT*-AY or *toxT*-AF alleles promoted CT and TcpA expression in almost all of the El Tor biotype strains tested, except for the IB5230 strain, in which the virulence gene expression was abrogated by the introduction of the *toxT*-AY or *toxT*-AF alleles. The B33 strain expressed comparable amounts of CT and TcpA at 37 °C after the introduction of *toxT*-AY or *toxT*-AF alleles.

N16961 and T19479 Wave 1 El Tor biotype strains: Neither CT nor TcpA were produced in the N16961(*toxT*-SY), YJB003 (N16961-*toxT*-SF), or T19479 (*toxT*-SY) strains under any of the conditions tested, including AKI conditions (Figure 3A,B). YJB006 (T19479-*toxT*-SF) in LB medium at 30 °C expressed approximately 40% of the CT and 100% of the TcpA expressed by the control strain, while, in AKI conditions, expressed 40% of the CT and 50% of the TcpA compared to the control strain (Figure 3A,B). Both CT and TcpA expression were markedly increased (above 60% for CT and 100% for TcpA, compared to the control) by the introduction of the *toxT*-AY (DHL008) and *toxT*-AF (DHL009) alleles in the N16961 strain at 30 °C (Figure 3A). No virulence genes were expressed at 37 °C. Virulence gene expression, especially of TcpA, was also significantly enhanced by the introduction of the *toxT*-AY (DHL011) or *toxT*-AF alleles (DHL012) in the T19479 strain at 30 °C (Figure 3B). Substantial amounts of TcpA were expressed by the DHL012 strain (above 80% of the control) under AKI conditions and when culturing in LB medium at 37 °C, while CT expression was only marginally increased (Figure 3B).

B33: The B33 strain was isolated during cholera outbreaks in 2002–2004 in Mozambique [39,40], and has been classified as a Wave 2 atypical El Tor strain [28]. Most of the Wave 2 atypical El Tor strains contain a tandem repeat of CTX-2 (*rstR* and *ctxB* are classical types) on Chromosome 2, while TLC (toxin-linked cryptic), RS1, and/or CTX-1 have been integrated into Chromosome 1 [41]. The B33 strain, in particular, does not contain any of these elements on Chromosome 1 [5]. The B33 strain originally contained the *toxT*-SY allele and expressed more CT at AKI conditions than the control, however, not when cultured in LB medium (Figure 3C). The introduction of the *toxT*-SF, *toxT*-AY, or *toxT*-AF alleles increased CT expression to as much as 200% of the control values when cultured in LB medium at 30 °C or under AKI conditions (Figure 3C). Moreover, approximately 150% of the control CT was expressed in isogenic derivatives of B33 that contained the *toxT*-AY (DHL017) or *toxT*-AF alleles (DHL018) when cultured in LB medium at 37 °C (Figure 3C). TcpA expression was also stimulated by the introduction of *toxT*-SF, *toxT*-AY, or *toxT*-AF alleles (Figure 3C).

MG116025: The MG116025 strain that originally contained the *toxT*-SF allele has been categorized as a Wave 2 atypical El Tor strain [28]. The *toxT*-SF allele was first identified in this strain because this strain could be transduced by CTXΦ by producing functional TCP when cultured in LB medium at 30 °C [23]. The introduction of the *toxT*-SY allele (YJB015) abolished the expression of CT and TcpA [25]. The expression of virulence genes was enhanced by the introduction of the *toxT*-AY (DHL014) or *toxT*-AF (DHL015) allele into MG116025 cultured under AKI conditions, in LB at 30 °C, and even in LB 37 °C (Figure 3D).

IB5230: The IB5230 strain was isolated during the 2010 cholera outbreak in Haiti and has been described as a hypervirulent *V. cholerae* strain, due to the elevated production of CT and other virulence-related factors [22,31]. Neither CT nor TcpA were expressed by the *toxT*-SY allele in IB5230 at 30 °C, while CT and TcpA were approximately 45% of the control values when this strain was cultured under AKI conditions (Figure 3E). Moreover, small amounts of CT (20% of the control) were produced at 37 °C, as previously reported [25]. The introduction of the *toxT*-SF allele remarkably stimulated virulence gene expression in the IB5230 strain when cultured at 30 °C and under AKI conditions. Unlike all of the other strains examined in this study, the introduction of the *toxT*-AY (DHL020) and *toxT*-AF (DHL021) alleles in this strain entirely abrogated virulence gene expression under all culture conditions (Figure 3E).

Our results indicate that ToxT production mediated by the ToxR regulon can occur in El Tor biotype *V. cholerae* strains after minimal stimulation (LB medium, shake culture at 30 °C) similar to the classical biotype strains. These findings imply that the regulation of the ToxT transcription factor and the expression of the virulence genes in *V. cholerae* may be more complex than previously believed.

## 3. Discussion

Our current understanding of the toxigenesis of *V. cholerae* has largely been obtained from studies of virulence gene expression under laboratory culture conditions; however, the induction of virulence gene expression in vivo infection is often inconsistent with the laboratory culture results. Classical biotype strains have been shown to produce CT and TCP at 30 °C, but not at 37 °C, under typical laboratory culture conditions, but they nonetheless provoke disease in the 37 °C environment of the human small intestine [32,42]. Special culture conditions have been developed to induce virulence gene expression in El Tor biotype strains—these include AKI culture conditions, increasing the surface/volume ratio and controlling the O_2_/CO_2_ ratio—that do not occur in the intestinal environment [12,19,20,43]. Moreover, these in vitro culture conditions are not generally applicable for *V. cholerae* strains, and the expression of virulence genes differs between strains, even within the same biotypes [21,27,38,44].

ToxT has been shown to directly bind to the promoters of virulence genes in *V. cholerae* [35,45]. The ToxT protein comprises 276 amino acids and belongs to the AraC/XylS-type transcriptional regulator family [13,46,47]. A conserved 107-amino acid C-terminal domain (CTD, amino acids 170–276) is the DNA-binding domain, while the N-terminal domain (NTD) plays roles in responses to environmental stimuli and perhaps in dimerization [48,49,50].

While *ctxB* and *tcpA* of *V. cholerae* are biotype-specific, *toxT* has been shown to be identical—*toxT*-SY—among *V. cholerae* strains [35]. *V. cholerae* strains that contain the *toxT*-SY allele have been shown to produce CT and TcpA under virulence-gene-inducing culture conditions (i.e., agglutinating conditions for classical biotype strains and AKI conditions for El Tor biotype strains); however, some *V. cholerae* strains do not produce CT or TcpA under these culture conditions [27,38].

Although most *V. cholerae* strains contain the *toxT*-SY allele regardless of biotype, two clinically isolated strains contain variant *toxT* alleles—the *toxT*-AY allele in a classical biotype strain 569B and the *toxT*-SF allele in the atypical El Tor biotype strain MG116025 [23]. These two strains produce CT and TcpA under simple culture conditions. We have previously reported that the replacement of the *toxT*-SY allele by *toxT*-SF facilitated CT and TcpA production in selected *V. cholerae* strains [27]. Moreover, we constructed the *toxT*-AF variant allele by replacing the Tyr residue at the 139th amino acid position with Phe in the 569B strain and found that the virulence gene expression was enhanced [27].

Alterations of the transcriptional activator function of ToxT by the scanning Ala mutagenesis have been reported [48]. The substitution of Ser at the 65th amino acid position of ToxT by Ala promoted 40% more transcription of a *lacZ* reporter gene linked to the *ctxA* promoter in a reporter strain; however, CT and TCP production by this variant ToxT in a *V. cholerae* strain was not tested [48]. Most of all, alterations of the transcriptional activator function of ToxT have not previously been studied extensively, especially in El Tor biotype strains.

In the present study, we constructed isogenic derivatives of nine well-established *V. cholerae* strains by introducing alternative *toxT* alleles and examined the stimulation of virulence gene expression under simple culture conditions. We anticipated that a particular ToxT allele might consistently stimulate the production of CT and TCP in *V. cholerae* strains.

Under laboratory culture conditions, a culture temperature of 30 °C seems to be optimal to trigger CT/TCP production in many *V. cholerae* strains, although certain El Tor biotype strains, especially atypical El Tor strains, could produce CT and TcpA at 37 °C in LB medium by a specific *toxT* allele. In many *V. cholerae* strains examined in this study, the replacement of Ser-65 by Ala promoted transcription of virulence genes under laboratory culture conditions, except in the IB5230 strain, in which the production of CT and TcpA was abolished by the same substitution.

Our results indicated that no specific *toxT* allele could consistently stimulate virulence gene expression in *V. cholerae* strains, even within the same biotype. Rather, each *toxT* allele showed strain-specific transcriptional activator functions among *V. cholerae* strains, suggesting that the regulation of virulence gene expression may involve more than just the production of ToxT by the ToxR regulon.

Structural differences among the *toxT* variants could be considered as one such additional modifier of the transcriptional activator functions of ToxT. Unsaturated fatty acids, such as linoleic acid, have been shown to inhibit transcriptional activation of virulence genes by ToxT [49,51], and structural analyses have suggested an association of cis-palmitoleate with ToxT [52]. We found that transcriptional activator functions of variant *toxT* alleles in some strains were differently modulated by unsaturated fatty acids (such data will be presented elsewhere). Virulence gene expression may also be modified by the rate of proteolysis of ToxT after the activation of virulence genes, and the susceptibility to proteolysis may, in turn, be influenced by point mutations at the 65th and 139th amino acid positions of *toxT* [35].

Differences in carbohydrate metabolism among the *V. cholerae* strains may also influence the virulence gene expression by *toxT* alleles. While Wave 1 El Tor biotype strains contain a functional neutral fermentation pathway, classical biotype strains lack the capacity for neutral fermentation [53], and atypical El Tor strains have also lost neutral fermentation due to a single nucleotide deletion mutation in the VC1589 gene encoding acetolactate decarboxylase [26]. Moreover, the restoration of the neutral fermentation pathway disrupted CT production in the hypervirulent IB5230 strain [25]. The regulation of virulence gene expression in *V. cholerae* remains to be fully elucidated.

The DNA sequences of cholera toxin promoter are identical among *V. cholerae* strains, except for the number of heptad repeats (5′ TTTTGAT 3′), which have been identified as binding sites for ToxR, ToxT, or H-NS [54]. *ctxAB* promoters of Cairo50, N16961, T19479, and B33 contain four heptad repeats; *ctxAB* promoters of Cairo48, MG116025, and IB5230 contain five heptad repeats; and *ctxAB* promoters of O395 and 569B contain seven and eight heptad repeats, respectively. Classical and El Tor biotype strains contain biotype-specific sequences in the promoter region of *tcpA* [51]. Both *ctxAB* and *tcpA* promoters contain two toxboxes (13-bp degenerate DNA sequence) similar to other accessory virulence genes that are directly controlled by ToxT [55]. The potential effects of the variations in the DNA sequence of these promoters on virulence gene expression by variant ToxT remain to be further examined.

Last, but not least, our results indicate that the expression of virulence genes can be induced in *V. cholerae* strains under relatively simple laboratory culture conditions. Moreover, under these conditions, strains such as B33 were able to express as much CT and TcpA as the hypervirulent IB5230 strain. Additional investigations are needed in order to compare virulence gene expression under laboratory culture conditions with in vivo virulence of *V. cholerae* strains.

## 4. Materials and Methods

### 4.1. Bacterial Strains

The bacterial strains used in this study are listed in Table 2.

### 4.2. Bacterial Culture

The bacterial strains were inoculated onto plates containing LB medium and streptomycin at a final concentration of 100 μg/mL. Single colonies were selected and inoculated into 3 mL of liquid LB medium and cultured in a shaking incubator o/n. The next day, 1 mL of the overnight culture was used to inoculate 10 mL of fresh LB medium to prepare a seed culture. The seed culture was shake-cultured at 30 °C or 37 °C for 8 h. Then, 100 μL of the seed culture was inoculated into 10 mL of fresh LB medium (pre-warmed at 30 °C or 37 °C) and cultured for 16 h. To culture the El Tor biotype strains in AKI conditions, the seed culture was shake-cultured at 37 °C for 4 h and inoculated in 10 mL of AKI broth (1.5% Bacto peptone, 0.4% yeast extract, 0.5% NaCl, and 0.3% NaHCO_3_) in a 15 mL round-bottom tube at 10^5^ CFU/mL, then incubated without shaking for 4 h. Then, the whole culture was transferred into a 250 mL flask and shake-cultured for 16 h.

After 16 h of main culture, bacterial cell numbers in the culture were counted. Whole cultures (for western blotting of secreted and intracellular CTB) and harvested cells (for western blotting of TcpA) were prepared for SDS-PAGE separately, and approximately 10^7^ cells were analyzed for SDS-PAGE and western blotting.

### 4.3. toxT Allele Exchange

Allele exchange at the 139th amino acid position: Two 843 bp DNA fragments, encompassing the 50 nucleotides upstream of the translation start codon to nucleotide 793 of *toxT*, were amplified by PCR using primers toxT-XbaIF: CCG GCC TCT AGA TAC GTG GAT GGC TCT CTG CG and toxT-SacIR: CCG GCC GAG CTC CAC TTG GTG CTA CAT TCA from the genomic DNA of *V. cholerae* strains MG116025 and N16961, respectively. The DNA fragments were inserted into a suicide plasmid, pCVD442, to construct pCVD-toxT-SF and pCVD-toxT-SY, respectively. The SNP at nucleotide 416 (A416 in N16961 and T416 in MG116025) lies in the center of these fragments. The *toxT*-SF allele of the MG116025 strain was replaced by the *toxT*-SY allele of the N16961 strain by the allelic exchange method, and, similarly, the *toxT*-SY alleles of the other strains were replaced with the *toxT*-SF allele of the MG116025 strain [23,25]. The 569B classical biotype strain contained another variant of *toxT*—*toxT*-AY—and the pCVD-toxT-SF was conjugally transferred to 569B to construct a variant of 596B that contains *toxT*-AF allele by allelic exchange. The replaced allele of *toxT* after the excision of the pCVD-442 suicidal vector was validated by Sanger sequencing.

Allele exchange at the 65th amino acid position: A set of allele exchange plasmids was used to construct variants at the 65th and 139th amino acid positions of *toxT*. Four DNA fragments (1308 bp), encompassing from the 709th nucleotide of *tcpF* to the 793rd nucleotide of *toxT,* were PCR-amplified by using a primer pair TcpF-XbaIF (GGG TCT AGA GAA TTA AGT AAG CAC GGG TA) and toxT-SacIR (CCG GCC GAG CTC CAC TTG GTG CTA CAT TCA) from the N16961, YJB003 (N16961-toxT-SF), 569B, and EJK007 (569B-toxT-AY) strains. These 4 DNA fragments were subcloned in a pCVD442 suicidal plasmid to construct pCVD-toxT-Out-SY, pCVD-toxT-Out-SF, pCVD-toxT-Out-AY, and pCVD-toxT-Out-AF. Then, pCVD-toxT-Out-SY and pCVD-toxT-Out-SF were conjugally transferred to the 569B (*toxT*-AY) and EJK007 (*toxT*-AF) strains, respectively, and 569B-toxT-SY and 569B-toxT-SF were constructed by allelic exchange. Similarly, pCVD-toxT-Out-AY and pCVD-toxT-Out-AF were conjugally transferred to other strains to construct isogenic variants that contained *toxT*-AY and *toxT*-AF alleles. The excision of the suicidal vector was screened on a sucrose-containing plate and the DNA sequence of the variant *toxT* was confirmed by Sanger sequencing.

### 4.4. Construction of toxT-Deleted (ΔtoxT) Strains

A 635 bp DNA fragment encompassing from the 709th nucleotide of *tcpF* to the 120th nucleotide of *toxT* was PCR-amplified using a primer pair TcpF-KpnIF: GGG GGT ACC GAA TTA AGT AAG CAC GGG TA and TcpF-BamHIR: CCC GGA TCC TGC AAT TCC ACT ATC TAT CCA from the genomic DNA of *V. cholerae* strain N16961 and inserted into the KpnI and BamHI sites of pUC18 to construct pUC-tcpF. A 660 bp DNA fragment encompassing from the 616th nucleotide of *toxT* to the 435th nucleotide of *tcpJ* was PCR-amplified using a primer pair TcpJ-BamHIF: GGG GGA TCC CTA GAG TCT CGA GGA GTA AAG and TcpJ-PstIR: CCC CTG CAG GCT GCA GAT AAA TAA ATG CC from the genomic DNA of the N16961 strain and inserted into the BamHI and PstI sites of pUC-tcpF to construct pUC-tcpF-tcpJ. A primer pair TcpF-XbaIF: GGG TCT AGA GAA TTA AGT AAG CAC GGG TA and TcpH-SacIR: CCC GAG CTC GCT GCA GAT AAA TAA ATG CC was used to PCR-amplify a 1301 bp DNA fragment from pUC-tcpF-tcpJ, and this DNA fragment was inserted into the pCVD442 suicidal plasmid to construct pCVD-del-toxT. pCVD-del-toxT was conjugally transferred to *V. cholerae* strains to construct *toxT*-deleted (Δ*toxT*) isogenic strains. The deletion of the internal fragment of *toxT* after the excision of the suicidal vector was validated by Sanger sequencing. In Δ*toxT* strains, 165 internal amino acids (from the 41st to 205th amino acids) of ToxT are deleted. The isogenic variant sets of the bacterial strains are shown in Table 2.

### 4.5. Construction of pBAD-toxT-SY

An 851 bp DNA fragment (including 831 bp of the toxT ORF, 18 bp coding for 6 His residues at the C-terminus, and 2 bp between the first and second amino acid position to adjust the reading frame) was PCR-amplified using a primer set toxT-NcoIF: GGG CCA TGG CCA TTG GGA AAA AAT CTT TTC AAA and toxT-SalIHis R: CCG GTC GAC TTA *GTG GTG GTG GTG GTG GTG* TTT TTC TGC AAC TCC TGT CAA CA (His-tag is shown in italics) from the genomic DNA of the O395. This DNA fragment was digested with NcoI/SalI and inserted into the same R.E sites of pBAD24 [29] to construct pBAD-toxT-SY. *E. coli* DH5α and O395-Δ*toxT* were transformed with pBAD-toxT-SY. _L_-arabinose was added to the bacterial culture at a final concentration of 0.2% for the induction of expression of ToxT from the P_BAD_ promoter. The expression of ToxT was confirmed by western blot analysis using anti-His-tag (Cell Signaling, Danvers, MA, USA).

### 4.6. SDS-PAGE and Western Blotting

The bacterial cells for the western blot analysis of TcpA or bacterial culture for the western blot analysis of CTB (approximately 10^7^ cells/well) were analyzed using 12 or 15% SDS-PAGE. For western blotting, proteins separated by SDS-PAGE were transferred onto a nitrocellulose membrane. The membrane was blocked for one hour at room temperature in TBS blocking buffer. Primary antibodies (anti-TcpA (a generous gift from Dr. W. F. Wade, Dartmouth University, Hanover, NH, USA [46]), and anti-CTB (ab34992, Abcam, Cambridge, UK) were diluted 1:10,000) were added to the membrane and incubated overnight at 4 °C. The membrane was then washed with TBST, and a secondary antibody (goat anti-rabbit IgG (diluted 1:20,000) or goat anti-mouse IgG (diluted 1:5000)) was added. Western blot images were analyzed using an Odyssey CLx imaging system (LI-COR Biosciences, Lincoln, NE, USA). The western blot band intensities representing TcpA and CTB were quantified using the ImageJ gel analysis program and LI-COR Odyssey software.

## Figures and Tables

**Table 1:** Amino acid variations of *toxT* alleles introduced into the *V. cholerae* strains in this study.

*toxT* Allele Found in	*toxT* Amino Acid Position		Allele Name
	65	139	
*V. cholerae* strains	S	Y	*toxT*-SY
MG116025	S	F	*toxT*-SF
569B	A	Y	*toxT*-AY
569B + MG116025	A	F	*toxT*-AF

**Table 2:** Bacterial strain list.

Strains	*toxT* Genotype	Genome Information and References	Strains	*toxT* Genotype	Genome Information and References
Classical biotype			El Tor Biotype		
O395	O395-*toxT*-SY (65S, 139Y)	CP000626/CP000627 [28]	N16961	N16961-*toxT*-SY	AE003852/AE003853 [29]
YJB001	O395-*toxT*-SF (65S, 139F)	[25]	YJB003	N16961-*toxT*-SF	[25]
EJK008	O395-*toxT*-AY (65A, 139Y)	This study	DHL008	N16961-*toxT*-AY	This study
EJK009	O395-*toxT*-AF (65A, 139F)	This study	DHL009	N16961-*toxT*-AF	This study
EJK010	O395-Δ*toxT*	This study	DHL010	N16961-Δ*toxT*	This study
569B	569B-toxT-AY	DADXPZ010000000	T19479	T19479-*toxT*-SY	ERS013250 [28]
EJK007	569B-*toxT*-AF	[27]	YJB006	T19479-*toxT*-SF	[25]
EJK011	569B-*toxT*-SY	This study	DHL011	T19479-*toxT*-AY	This study
EJK012	569B-*toxT*-SF	This study	DHL012	T19479-*toxT*-AF	This study
EJK013	569B-Δ*toxT*	This study	DHL013	T19479-Δ*toxT*	This study
Cairo50	Cairo50-*toxT*-SY	ERS013165 [28]	MG116025	MG116025-*toxT*-SF	ERS013135 [28]
EJK004	Cairo50-*toxT*-SF	[27]	YJB015	MG116025-*toxT*-SY	[25]
EJK014	Cairo50-*toxT*-AY	This study	DHL014	MG116025-*toxT*-AY	This study
EJK015	Cairo50-*toxT*-AF	This study	DHL015	MG116025-*toxT*-AF	This study
EJK016	Cairo50-Δ*toxT*	This study	DHL016	MG116025-Δ*toxT*	This study
Cairo48	Cairo48-*toxT*-SY	ERS013171 [28]	B33	B33-*toxT*-SY	ACHZ00000000 [30]
EJK003	Cairo48-*toxT*-SF	[27]	YJB009	B33-*toxT*-SF	[25]
EJK017	Cairo48-*toxT*-AY	This study	DHL017	B33-*toxT*-AY	This study
EJK018	Cairo48-Δ*toxT*	This study	DHL018	B33-*toxT*-AF	This study
			DHL019	B33-Δ*toxT*	This study
			IB5230	IB5230-*toxT*-SY	AELH00000000.1 [31]
			YJB020	IB5230-*toxT*-SF	[25]
			DHL020	IB5230-*toxT*-AY	This study
			DHL021	IB5230-*toxT*-AF	This study
			DHL022	IB5230-Δ*toxT*	This study

## Data Availability

Not applicable.

## Supplementary Materials

The following supporting information can be downloaded at: https://www.mdpi.com/article/10.3390/toxins15080507/s1, Figure S1. Coomassie brilliant blue-stained SDS-PAGE of the isogenic derivatives of the classical biotype strain O395. (A) Lane 1: *E. coli* DH5α harboring pBAD-toxT cultured in the presence of 0.2% arabinose in LB medium at 37 °C, lane 2: *E. coli* DH5α harboring pBAD-toxT cultured in the absence of 0.2% arabinose in LB medium at 37 °C, lane 3: EJK010 (O395-ΔtoxT) that contained pBAD-toxT cultured at 30 °C in the presence of 0.2% arabinose in LB medium, lane 4: EJK010 (O395-ΔtoxT) that contained pBAD-toxT cultured at 37 °C in the presence of 0.2% arabinose in LB medium, lane 5: EJK010 (O395-ΔtoxT) that contained pBAD-toxT cultured in the absence of 0.2% arabinose in LB medium at 30 °C, and lane 6: EJK010 (O395-ΔtoxT) that contained pBAD-toxT cultured in the absence of 0.2% arabinose in LB medium at 37 °C. (B) Lane (C): IB5230 cultured in AKI broth at 37 °C, lane 1: EJK010 cultured in LB medium at 30 °C, lane 2: EJK010 cultured in LB medium at 37 °C, lane 3: EJK010 that contained pBAD-toxT cultured in LB medium at 30 °C in the presence of 0.2% arabinose, lane 4: EJK010 that contained pBAD-toxT cultured in LB medium at 37 °C in the presence of 0.2% arabinose, lane 5: EJK010 that contained pBAD-toxT cultured in LB medium at 30 °C in the absence of 0.2% arabinose, and lane 6: EJK010 that contained pBAD-toxT cultured in LB medium at 37 °C in the absence of 0.2% arabinose; Figure S2. CT and TCP expression by toxT variants in classical biotype strain analyzed by western blotting. Representative western blot images of expression of CTB (indicated in the red box in the left panel) and TcpA (indicated in the blue box in the right panel) of (A) O395, (B) 569B, (C) Cairo48, and (D) Cairo50, cultured in LB medium at 30 °C (LB 30), agglutinating conditions (Agg.), and LB medium at 37 °C (LB 37); Figure S3. CT and TCP expression by toxT variants in El Tor biotype strain analyzed by western blotting. Representative western blot images of the expression of CTB (indicated in the red box in the left panel) and TcpA (indicated in the blue box in the right panel) of (A) N16961, (B) T19479, (C) B33, (D) MG116025, and (E) IB5230, cultured in LB medium at 30 °C (LB 30), AKI conditions (AKI con), and LB medium at 37 °C; Table S1. CT and TCP expression by toxT variants in classical biotype strains of *V. cholerae*. Relative expression levels (mean ± standard deviation) of CT and TCP compared to the positive control (IB5230 cultured in AKI broth at 37 °C) as 100% are displayed; Table S2. CT and TCP expression by toxT variants in El Tor biotype strains of *V. cholerae*. Relative expression levels (mean ± standard deviation) of CT and TCP compared to the positive control (IB5230 cultured in AKI broth at 37 °C) as 100% are displayed.
