# Supplementary material for: Expression of Cholera Toxin (CT) and the Toxin Co-Regulated Pilus (TCP) by Variants of ToxT in Vibrio cholerae Strains
Source: Toxins (Basel). 2023 Aug 17;15(8):507. doi: 10.3390/toxins15080507 (PMC10467113; doi:10.3390/toxins15080507)
Supplement: Supplementary file 1 [file toxins-15-00507-s001.zip › toxins-2528238-supplementary.pdf]

Supplementary Materials: Expression of Cholera Toxin (CT) and the Toxin Co-Regulated Pilus (TCP) by Variants of ToxT in *Vibrio cholerae* Strains

Donghyun Lee, Hunseok Choi, Seonghyeon Son, Jonghyun Bae, Jayun Joo, Dong Wook Kim and Eun Jin Kim

Fig.S1.

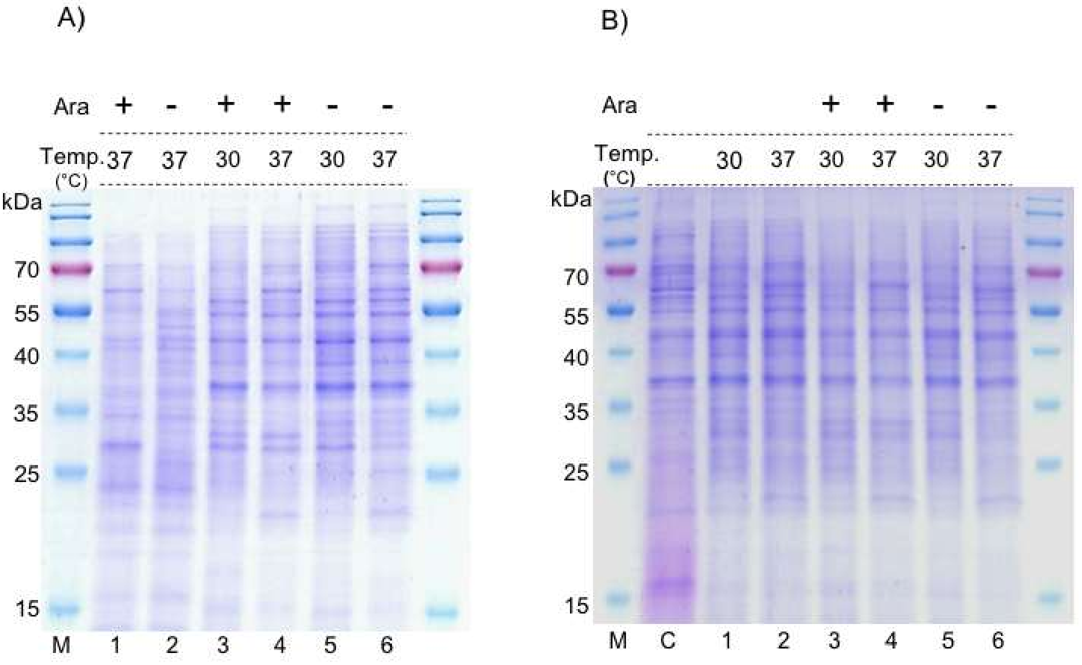

Figure S1. Coomassie brilliant blue-stained SDS-PAGE of the isogenic derivatives of the classical biotype strain O395. (A) Lane 1: *E. coli* DH5α harboring pBAD-toxT cultured in the presence of 0.2% arabinose in LB medium at 37 °C, lane 2: *E. coli* DH5α harboring pBAD-toxT cultured in the absence of 0.2% arabinose in LB medium at 37 °C, lane 3: EJK010 (O395-ΔtoxT) that contained pBAD-toxT cultured at 30 °C in the presence of 0.2% arabinose in LB medium, lane 4: EJK010 (O395-ΔtoxT) that contained pBAD-toxT cultured at 37 °C in the presence of 0.2% arabinose in LB medium, lane 5: EJK010 (O395-ΔtoxT) that contained pBAD-toxT cultured in the absence of 0.2% arabinose in LB medium at 30 °C, and lane 6: EJK010 (O395-ΔtoxT) that contained pBAD-toxT cultured in the absence of 0.2% arabinose in LB medium at 37 °C. (B) Lane (C): IB5230 cultured in AKI broth at 37 °C, lane 1: EJK010 cultured in LB medium at 30 °C, lane 2: EJK010 cultured in LB medium at 37 °C, lane 3: EJK010 that contained pBAD-toxT cultured in LB medium at 30 °C in the presence of 0.2% arabinose, lane 4: EJK010 that contained pBAD-toxT cultured in LB medium at 37 °C in the presence of 0.2% arabinose, lane 5: EJK010 that contained pBAD-toxT cultured in LB medium at 30 °C in the absence of 0.2% arabinose, and lane 6: EJK010 that contained pBAD-toxT cultured in LB medium at 37 °C in the absence of 0.2% arabinose.

Fig.S2.

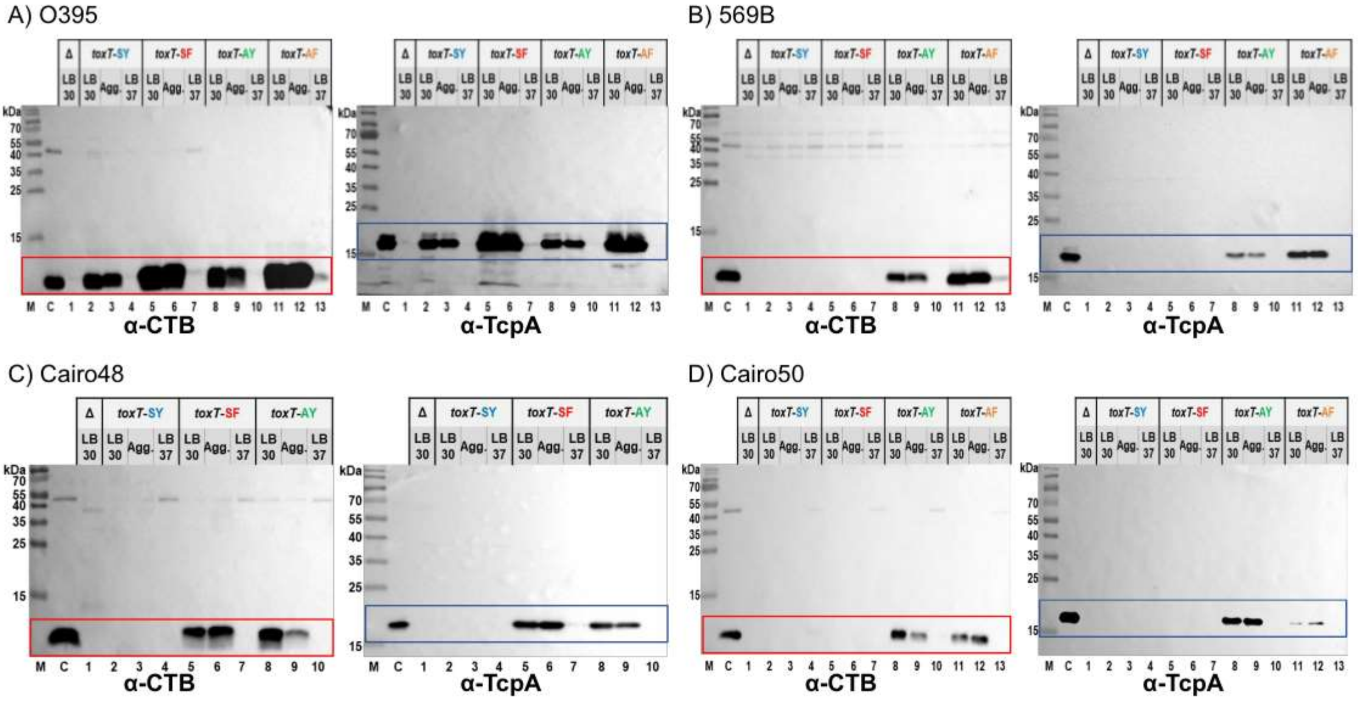

Figure S2. CT and TCP expression by toxT variants in classical biotype strain analyzed by western blotting. Representative western blot images of expression of CTB (indicated in the red box in the left panel) and TcpA (indicated in the blue box in the right panel) of (A) O395, (B) 569B, (C) Cairo48, and (D) Cairo50, cultured in LB medium at 30 °C (LB 30), agglutinating conditions (Agg.), and LB medium at 37 °C (LB 37).

Fig.S3.

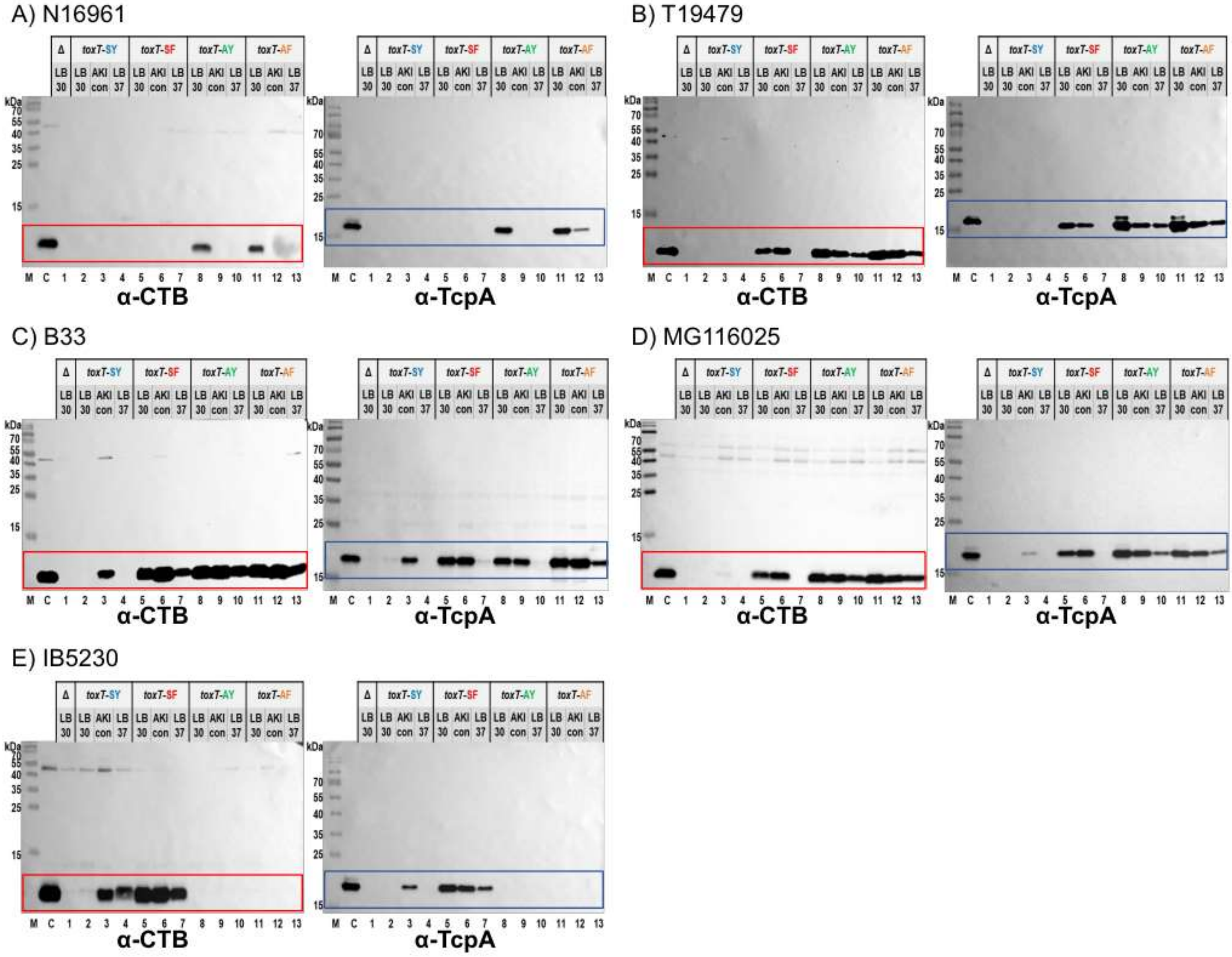

.

Figure S3. CT and TCP expression by toxT variants in El Tor biotype strain analyzed by western blotting. Representative western blot images of the expression of CTB (indicated in the red box in the left panel) and TcpA (indicated in the blue box in the right panel) of (A) N16961, (B) T19479, (C) B33, (D) MG116025, and (E) IB5230, cultured in LB medium at 30 °C (LB 30), AKI conditions (AKI con), and LB medium at 37 °C.

Table S1. CT and TCP expression by *toxT* variants in classical biotype strains of *V. cholerae*. Relative expression levels (mean ± standard deviation) of CT and TCP compared to the positive control (IB5230 cultured in AKI broth at 37 °C) as 100% are displayed.

**Strains**	**CT**		**TCP**	
Classical Biotype	LB 30°C	LB 37°C	LB 30°C	LB 37°C
O395 (*toxT*-SY)	136(±47)	0	101(±24)	0
YJB001 (*toxT*-SF)	310(±48)	24(±24)	204(±36)	10(±2)
EJK008 (*toxT*-AY)	90(±6)	0	86(±22)	0
EJK009(*toxT*-AF)	315(±67)	16(±5)	207(±23)	0
EJK010(Δ*toxT*)	0	0	0	0
569B (*toxT*-AY)	44 (±7)	0	44 (±7)	0
EJK007 (*toxT*-AF)	119 (±26)	0	119 (±26)	0
EJK011 (*toxT*-SY)	0	0	0	0
EJK012 (*toxT*-SF)	0	0	0	0
EJK013 (Δ*toxT*)	0	0	0	0
Cairo50 (*toxT*-SY)	0	0	0	0
EJK004 (*toxT*-SF)	0	0	0	0
EJK014 (*toxT*-AY)	40(±18)	0	51(±13)	0
EJK015 (*toxT*-AF)	34(±12)	1	1	0
EJK016 (Δ*toxT*)	0	0	0	0
Cairo48 (*toxT*-SY)	0	0	0	0
EJK003 (*toxT*-SF)	86 (±29)	0	99 (±6)	0
EJK017 (*toxT*-AY)	83 (±20)	0	96 (±7)	0
EJK018 (Δ*toxT*)	0	0	0	0

Table S2. CT and TCP expression by *toxT* variants in El Tor biotype strains of *V. cholerae.* Relative expression levels (mean ± standard deviation) of CT and TCP compared to the positive control (IB5230 cultured in AKI broth at 37 °C) as 100% are displayed.

**Strains**	**CT**			**TCP**		
El Tor Biotype	LB 30°C	AKI condition	LB 37°C	LB 30°C	AKI condition	LB 37°C
N16961 (*toxT*-SY)	0	0	0	0	0	0
YJB003 (*toxT*-SF)	0	0	0	0	0	0
DHL008 (*toxT*-AY)	78 (±28)	0	0	101 (±12)	0	0
DHL009 (*toxT*-AF)	70 (±29)	0	0	118 (±8)	20 (±8)	2 (±3)
DHL010 (Δ*toxT*)	0	0	0	0	0	0
T19479 (*toxT*-SY)	0	0	0	0	0	0
YJB006 (*toxT*-SF)	37 (±17)	43 (±6)	0	98 (±8)	51 (±5)	0
DHL011 (*toxT*-AY)	62 (±30)	64 (±6)	21 (±17)	161 (±22)	73 (±10)	28 (±22)
DHL012 (*toxT*-AF)	61 (±37)	89 (±15)	60 (±23)	171 (±23)	114 (±3)	88 (±4)
DHL013 (Δ*toxT*)	0	0	0	0	0	0
MG116025 (*toxT*-SF)	55 (±16)	39 (±28)	0	91 (±9)	84 (±23)	0
YJB015 (*toxT*-SY)	0	0	0	0	14 (±6)	0
DHL014 (*toxT*-AY)	126 (±31)	65 (±29)	30 (±13)	117 (±19)	102 (±8)	58 (±11)
DHL015 (*toxT*-AF)	78 (±63)	42 (±32)	25 (±25)	109 (±20)	88 (±12)	66 (±16)
DHL016 (Δ*toxT*)	0	0	0	0	0	0
B33 (*toxT*-SY)	0	105 (±59)	0	0	52 (±9)	0
YJB009 (*toxT*-SF)	111 (±42)	176 (±40)	43 (±15)	109 (±49)	103 (±41)	4 (±6)
DHL017 (*toxT*-AY)	174 (±51)	162 (±29)	106 (±21)	95 (±18)	72 (±21)	0
DHL018 (*toxT*-AF)	149 (±26)	183 (±45)	140 (±48)	127 (±36)	138 (±32)	65 (±11)
DHL019 (Δ*toxT*)	0	0	0	0	0	0
IB5230 (*toxT*-SY)	0	76 (±26)	40 (±6)	0	44 (±10)	0
YJB020 (*toxT*-SF)	103 (±2)	99 (±16)	54 (±29)	129 (±12)	104 (±12)	41 (±9)
DHL020 (*toxT*-AY)	0	0	0	0	0	0
DHL021 (*toxT*-AF)	0	0	0	0	0	0
DHL022 (Δ*toxT*)	0	0	0	0	0	0
